# Slowing Heart Rate Protects Against Pathological Cardiac Hypertrophy

**DOI:** 10.1093/function/zqac055

**Published:** 2022-11-01

**Authors:** Sonia Sebastian, Lee S Weinstein, Andreas Ludwig, Patricia Munroe, Andrew Tinker

**Affiliations:** William Harvey Heart Centre, Barts and The London School of Medicine and Dentistry, Queen Mary University of London, Charterhouse Square, London EC1M 6BQ, UK; Metabolic Diseases Branch, National Institute of Diabetes and Digestive and Kidney Diseases/National Institutes of Health, Building 10, Room 8C101, Bethesda, MD 20892-1752, USA; Institut fuer Experimentelle und Klinische Pharmakologie und Toxikologie, Universitaet Erlangen-Nuernberg, Fahrstr. 17, 91054 Erlangen, Germany; William Harvey Heart Centre, Barts and The London School of Medicine and Dentistry, Queen Mary University of London, Charterhouse Square, London EC1M 6BQ, UK; William Harvey Heart Centre, Barts and The London School of Medicine and Dentistry, Queen Mary University of London, Charterhouse Square, London EC1M 6BQ, UK

**Keywords:** heart rate, ventricular hypertrophy, sinoatrial node, stimulatory heterotrimeric G-protein

## Abstract

We aimed to determine the pathophysiological impact of heart rate (HR) slowing on cardiac function. We have recently developed a murine model in which it is possible to conditionally delete the stimulatory heterotrimeric G-protein (Gα_s_) in the sinoatrial (SA) node after the addition of tamoxifen using cre-loxP technology. The addition of tamoxifen leads to bradycardia. We used this approach to examine the physiological and pathophysiological effects of HR slowing. We first looked at the impact on exercise performance by running the mice on a treadmill. After the addition of tamoxifen, mice with conditional deletion of Gα_s_ in the SA node ran a shorter distance at a slower speed. Littermate controls preserved their exercise capacity after tamoxifen. Results consistent with impaired cardiac capacity in the mutants were also obtained with a dobutamine echocardiographic stress test. We then examined if HR reduction influenced pathological cardiac hypertrophy using two models: ligation of the left anterior descending coronary artery for myocardial infarction and abdominal aortic banding for hypertensive heart disease. In littermate controls, both procedures resulted in cardiac hypertrophy. However, induction of HR reduction prior to surgical intervention significantly ameliorated the hypertrophy. In order to assess potential protein kinase pathways that may be activated in the left ventricle by relative bradycardia, we used a phospho-antibody array and this revealed selective activation of phosphoinositide-3 kinase. In conclusion, HR reduction protects against pathological cardiac hypertrophy but limits physiological exercise capacity.

## Graphical Abstract

**Graphical Abstract:**
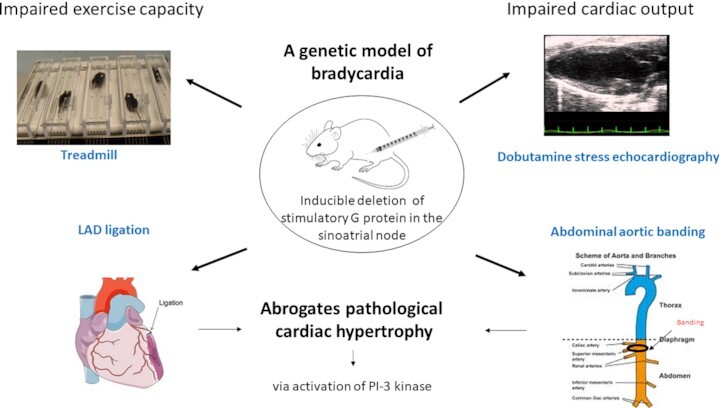

## Introduction

The resting heart rate (HR) like blood pressure is known to be associated with cardiovascular risk and outcomes. An increased HR is associated with increased mortality and this has been shown in a number of epidemiological studies.^1,2^ For example, in the Paris Prospective Study I, the risk of sudden death was increased over 3-fold in those with a resting HR above 75 beats-per-minute compared to those individuals who had one under 60 beats-per-minute.^3^ Furthermore, in various cardiovascular diseases including coronary artery disease and heart failure a selective intervention to reduce the HR may be beneficial.^4^ Ivabradine (IVB) is a clinically used drug that selectively blocks a nonselective cation current known as If (“funny” current) in the sinoatrial (SA) node, and as a result slows the HR without affecting inotropy and blood pressure as compared to the effects of beta blockers.^5,6^ In the SHIFT trial, the addition of IVB to standard treatment in chronic cardiac failure with reduced ejection fraction (EF) led to a significant reduction in the primary endpoints of hospitalizations for treatment and death.^7^ Furthermore, a lower HR clearly correlated with improved outcomes.^8^ However, it has been argued that IVB may directly interact with HCN channels in the ventricle particularly when affected by disease and its effects arise from this action and not a direct effect on HR per se.^9–12^. Specifically, the actions of IVB on If in the failing left ventricle may affect electrical remodeling and attenuate apoptosis and hypertrophy.^4^ It is also not clear in traditional epidemiological studies whether the association between HR and mortality is causal, and if so what the mechanism might be.

The stimulatory heterotrimeric G-protein (Gα_s_) subunit is ubiquitously expressed and mediates key signaling pathways. For example, in the heart the positive chronotropic and inotropic actions via the sympathetic nervous system are entrained by binding of noradrenaline and adrenaline to β adrenergic receptors and activation of Gα_s_. We have recently reported a murine model in which we can conditionally and selectively delete Gα_s_ in the SA node.^13^ We used a tamoxifen-inducible cre recombinase construct that was inserted into the hcn4 locus^14^ and crossed with Gα_s_-floxed mice in which loxP sites were placed upstream and downstream of exon 1.^13^ The administration of tamoxifen to these mice leads to a fall in HR, attenuation of the positive chronotropic effects of isoprenaline, and a reduction in normalized low frequency power in HR variability analysis. This approach has the advantage that it is genetic and not pharmacological in nature whilst still being selective and open to temporal manipulation via tamoxifen. Thus, we used these mice to investigate the benefits or otherwise of HR reduction in cardiac physiological and pathological processes.

## Methods

### Experimental Animals

Mice were maintained in an animal core facility under the United Kingdom Home Office guidelines relating to animal welfare compatible with Directive 2010/63/EU of the European Parliament. All maintenance, breeding, and procedures were covered by project licences PPL 70\7665 and PE9055EAD. All mice were kept in a temperature-controlled environment (21–23°C) with 12:12-h dark–light cycles. Animals were allowed access to standard rodent chow and water ad libitum. Mice were studied between 8 and 12 wk of age. A description of the targeting strategy to produce Gα_s_ (flx, flx) mice and the genotyping strategy has previously been published. ^13–15^ The littermate control mice were either cre + or wildtype genotype. There were no differences between these groups. Tamoxifen (Sigma) was freshly dissolved in sunflower oil (10 mg/mL) and 1 mg tamoxifen/25 g body weight was injected intraperitoneally (i.p.) on 5 consecutive days. The operator was blinded to the genotype of the mice when performing treadmill experiments, echocardiography, and surgical interventions. At the end of each study, animals were culled by exposing them in the CO_2_ chamber for 4 min and then by cervical dislocation before disposal. A mixture of sexes was used in this study (52 male and 64 female).

### Treadmill Studies

Mice were acclimatized on a treadmill (model Eco 3/6 from Columbus Instruments) prior to the experimentation. We followed an acute exercise protocol, where mice are run to exhaustion.^16^ Mice were encouraged to run on the treadmill belt setup at a speed of 7 m/min and then gradually increased to 9 m/min. This was repeated for 3 consecutive days a week, and then for the experimental day they were run on the treadmill starting with the speed of 9 m/min up to 13 m/min. Exhaustion time and distance covered were noted for each mouse. Exhaustion time was defined as greater than 5 s on the plate at the back of the treadmill without attempting to reengage the treadmill or spending greater than 50% of its time on the plate at the back of the treadmill.^17^

### Murine In Vivo Model of Myocardial Infarction

The myocardial infarction (MI) model employed for this study comprised of occlusion of left anterior descending coronary artery. The main surgical procedure is described by Fisher and Marber^18^ with modifications.^19^ All surgical procedures were conducted in a controlled sterile surgical theatre and aseptic operating practices were adhered to throughout the procedure. ECG was recorded throughout the procedure and monitored by Power lab coupled to chart 7.0. Temperature was monitored by a rectal thermoprobe and was maintained at 37°C ± 0.5. Artificial ventilation was provided throughout the main surgical procedure by means of a MiniVent mouse ventilator (HSE-HA MiniVent Mouse Ventilator # 730027 Harvard Apparatus, March-Hugstetten, Germany) Mice were anaesthetized with 4% isoflurane vaporized in oxygen and maintenance anaesthesia, via the ventilator, was supplied with 2% isoflurane and a tidal volume of 300 μL at 120 cycles/min. Mice were placed in the supine position with neck extended using a suture hooked around the incisor and secured using adhesive tape. Analgesia was provided by buprenorphine (Vetergesic 0.1 mg/kg intramuscular) at the start of the procedure. A small skin incision was made to the neck and the skin and submaxillary glands were gently teased apart to expose the trachea. The ventilation cannula was passed through the mouth into the trachea and successful intubation was confirmed once the cannula could be directly visualized inside trachea and further confirmed by observing regular chest movements upon ventilation. For access to the LAD, the mouse was repositioned by crossing the left leg over the body to aid access to the left third and fourth intercostal spaces. A skin incision was made laterally across the chest at the approximate position of the third and fourth intercostal space. The major and minor pectoral muscles were blunt dissected and reflected outward using hook stays. Dissection was performed by creating a small hole using sharp pointed scissors followed by insertion of a small animal retractor. Access to the LAD was facilitated by opening of the pericardium using blunt tweezers. LAD was under run by 8.0 prolene suture (#W2775 ETHICON), ideally 2 mm below the tip of the left atrium. The suture was double-knotted in order to occlude the LAD. Successful MI upon occlusion was confirmed primarily by myocardial color change from red to pink and ST segment elevation. Animals were allowed to recover for 2 wk and were then subjected to echocardiographic measurements.

### Tissue Processing and TTC Staining

After MI studies, the heart was excised, washed in cold phosphate buffered saline (PBS), frozen at −80 for 10 min, the atria were removed, and the heart was sectioned into 5 to 6 transverse slices from the apex to the base by placing it in a mould in the shape of the heart. The slices were arranged on a tissue culture dish in order from apex to base. The slices were then incubated with 2 mL of 1% 2,3,5-triphenyltetrasodium chloride (TTC #T8877 Sigma) in PBS for 30 min at 37°C, fixed in 4% formalin for 24 h, washed with PBS, and scanned. TTC stains viable myocardium brick red as it is reduced by NADH, whereas necrotic myocardium appears pale yellow.^20^ The infarct size was quantified by analyzing the areas of necrotic and total myocardium from the serial images of the slices in ImageJ.

### Histology

After MI, mouse hearts were collected and rinsed thoroughly in cold PBS to remove excess blood and fixed in 10% formalin for 24 h. After that, they were washed 3 times in PBS and stored in 70% ethanol before paraffin embedding. Paraffin-embedded myocardia were cut into 5-μm-thick sections and mounted on clear Plus microscope slides. For histological analysis, sections were stained for haematoxylin and eosin with automated Leica autostainer XL system (Leica Biosystems, UK), trichrome stain kit (Ab150686, Abcam, UK) to detect cardiac fibrosis (according to the manufacturer’s instructions).

### Wheat Germ Agglutinin Staining

After deparaffinization process of cardiac sections, they were rinsed thoroughly with 1X PBS and placed in dark humidified chamber. A volume of 50 µg/mL solution of wheat germ agglutinin (WGA) conjugated with CF-555 fluorescent dye (Cambridge Bioscience, UK) was applied to each section with a Pasteur pipette ensuring that the whole section area is covered with the solution and was incubated for 1 h at room temperature. After incubation, WGA solution was removed and then washed thoroughly with double distilled water to prevent the crystallization. Sections were than left to dry in the dark for 10 min. After that, sections were counterstained with DAPI for 5 min and mounted with Thermo Scientific Shandon EZ-Mount Mountant (Shandon, UK) and prepared for imaging. Images were obtained using Nanozoomer S60 slide scanner (Hamamatsu, Japan) using DAPI and red filters at 20x magnification and analyzed using the software NDP view2 (Hamamatsu). Extent of fibrosis and cross-sectional area was quantified using Image J software.^21^

### Abdominal Aortic Banding

The abdominal aorta, between the left and right renal arteries, is constricted during the surgery.^22^ Cardiac hypertrophy and remodeling can be observed 2 wk after the abdominal aortic constriction surgery.^18,23^ Mice were anaesthetized with 4% isoflurane vaporized in oxygen. Aseptic conditions were maintained throughout the procedure. Long-term maintenance anaesthesia, was supplied with 2% isoflurane and a tidal volume of 300 μL at 120 cycles/min. Mice were placed in a supine position on a surgery platform with a heating pad maintained at 37°C. Analgesia was provided by buprenorphine (Vetergesic 0.1 mg/kg intramuscular) at the start of the procedure. Hair was removed with a hair remover lotion and cleanly shaven abdomen was scrubbed with Betadine. An incision of 2 cm was made along the midline of the abdomen with a sterile scalpel, normal saline was used to keep digestive organs moist during the surgery. Digestive organs were carefully displaced to the side using cotton balls to expose the inferior vena cava that lies in the posterior peritoneal region. The abdominal aorta was identified and peritoneum was pierced with a pair of forceps to uncover the vessels beneath. The abdominal aorta adjacent to the renal arteries was isolated and an 8-cm-long 4–0 silk suture (ETHICON) was passed underneath the abdominal aorta, and banding was performed just above renal arteries (∼2 mm). A loose double knot was made with the suture; and a blunted and bent 22 G needle was placed inside the loop. The knot around the aorta and the needle was tightened, and then the needle was removed immediately to achieve a 0.7-mm diameter constriction. The abdominal cavity was closed with 6–0 absorbable suture material and subsequently the muscle or skin incisions with simple interrupted sutures. Animals were allowed to recover for 2 wk and were then subjected to echocardiographic measurements.

### Murine Echocardiography

Echocardiography was performed using a Vevo 770 and Vevo 3100 ultrasound system (Visual Sonics, Toronto, Canada) equipped with a real-time microvisualization scan probe head (RMV-707B) working at a frame rate of 100 frames per second and with a transducer, which has a central frequency of 30 MHz. Mice were anaesthetized with isoflurane, at a concentration of 4% (induction) and 1.5% (maintenance) in 100% oxygen. Each animal was placed on a heating table in a supine position with the extremities tied to the table through surgical tape. After the removal of hair from the chest, warm ultrasound gel (Aquagel lubricating gel from Parker Labs, New Jersey) was applied to the thorax surface to optimize the visualization of the cardiac chambers. Temperature was monitored by rectal probe and was maintained at 37°C ± 0.5 by adjusting the temperature on the heating table. 2D-guided left ventricular (LV) M-mode echocardiography is recorded from either the short-axis view and/or the long-axis view at the level of papillary muscles.

**Figure 1.**
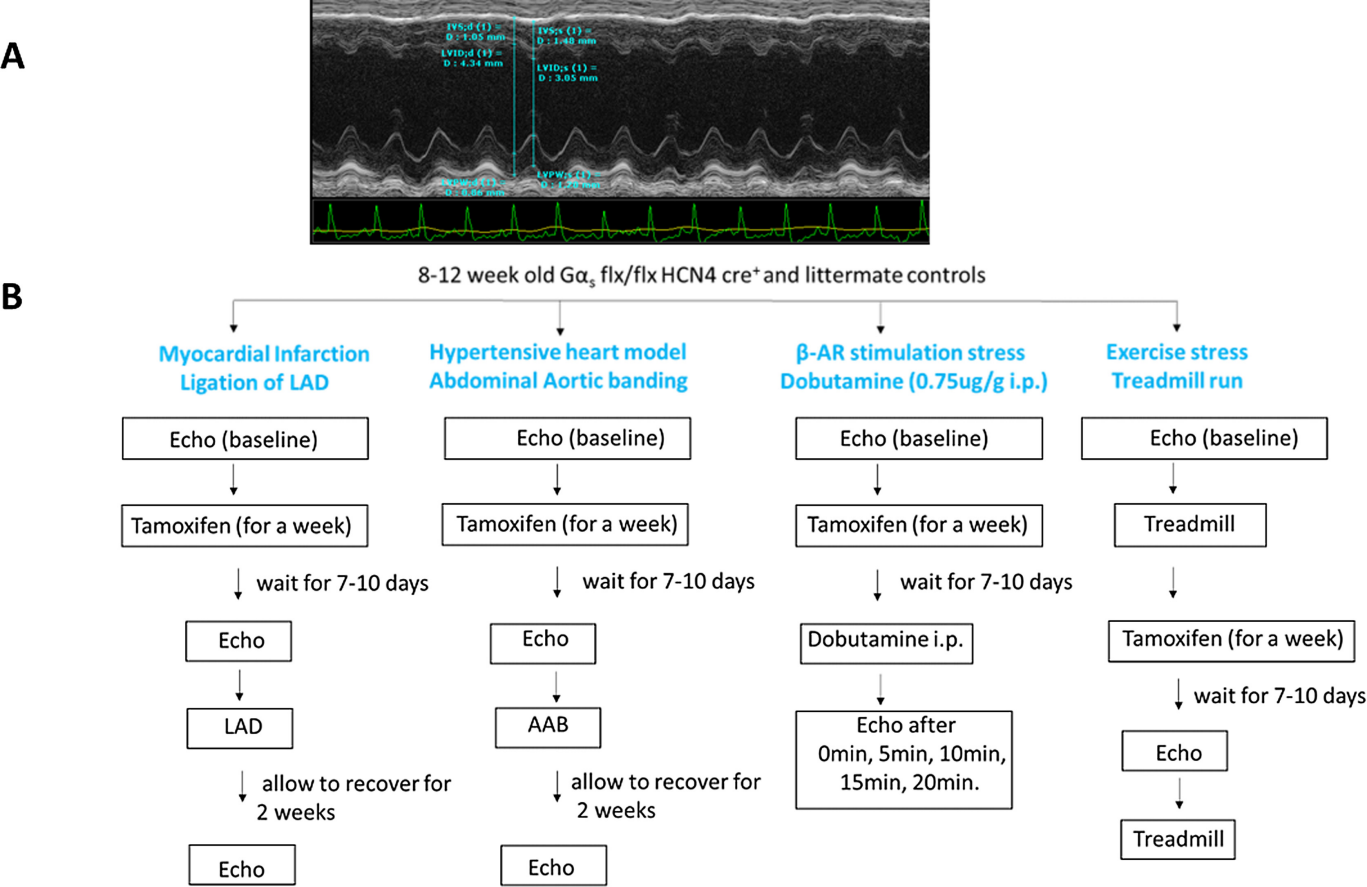
Wall dimension measurements on M-mode echocardiography and study design. (A) LV function was determined by echocardiographic measurements or wall dimensions obtained from transthoracic echocardiography in anaesthetized mice. (B) A schematic representation of the various experimental protocols.

Measurements were made using the leading edge to leading edge method according to the guidelines and standards of the American Society of Echocardiography.^24^ LV wall thickness is evaluated at the interventricular septum (IVS) and the posterior wall (LVPW). End-diastolic measurements [interventricular septal thickness diastolic (IVSd), left ventricular posterior wall thickness diastolic (LVPWd), and left ventricular internal dimension diastolic (LVIDd)] are obtained at the point of maximal LV diastolic dimension. LV end-systolic dimensions (IVSs, LVPWs, and LVIDs) are obtained at the time of most anterior systolic excursion of the LVPW associated with minimal chamber dimension. All LV dimensions are presented as the average of measurements using the leading-edge technique of 3–5 consecutive selected sinus beats. LV systolic function is evaluated from fractional shortening (FS%), EF (EF%), and cardiac output [CO, determined from HR and stroke volume (SV)]^25^ and calculated from M-mode-derived LV dimensions using the formulae below.^26–28^ The measurement of the standard parameters is illustrated in Figure 1(A).

SV = LVIDd^3^-LVIDs^3^; CO = HRxSV.
EF% = 100x[(LVIDd^3^-LVIDs^3^)/LVIDd^3^].
FS% = 100x[(LVIDd-LVIDs)/LVIDd].
LV mass = 1.05[(IVSd + LVIDd + PWD)^3^-LVIDd^3^.

### Dobutamine Stress Study

Dobutamine hydrochloride (DOB) powder (Sigma-Aldrich, product number D 0676) was dissolved in ddH_2_O by gently heating at 37°C for 15–20 min and vortexing to produce a 10 mg/mL stock solution. DOB working solution (0.1 μg/μL) was prepared by 1:10 dilution of the stock dilution in sterile 0.9% NaCl. Mice were characterized via baseline echocardiography and ECG, before receiving an i.p. single bolus injection of DOB at a dose of 0.75 μg/g body weight. The DOB dose (0.75 μg/g i.p.) was selected on the basis of preliminary experiments using 3 different doses (0.375, 0.75, or 1.5 μg/g i.p.), which revealed that 0.75 μg/g was sufficient to trigger a pronounced cardiac response in a cohort of adult C57/Bl6 male mice.^29^ Echocardiographic B- and M-Mode scans and pulmonary artery for pulse wave in long- and short-axis projections, pulmonary artery flow for pulse wave and ECG recordings were repeated immediately after the single bolus injection and then during 5, 10, 15, and 20 min until the peak HR response was reached and HR began to decline again. We show selected parameters before and at 20 min after DOB administration for simplicity of presentation. IVB (SML0281 Sigma Aldrich) was dissolved in PBS to 10 mg/mL stock and administered in a volume of 0.1 mL/10 g of body weight.^30^ Mice received an i.p. single bolus injection of IVB at a dose of 10 mg/kg body weight before and after tamoxifen. ECG recordings were repeated immediately after the single bolus injection and then during 5, 10, 15, and 20 min until the peak HR response was reached.

### Phospho Antibody Array

Antibody Microarray is a high-throughput ELISA based platform for efficient protein expression profiling, screening, and comparison between normal and treated samples. We used ERK phospho Antibody Array from Full Moon Bio Systems (PEK208) and array assay kit (KS02) and followed the manufacturer’s instruction for the extraction and detection of proteins and slides were shipped back to them for analysis. A total of 227 specific kinases were assayed. The median of 6 technical replicates was used for each sample\mouse. The pdf and excel spreadsheet provided by the company are provided as supplementary material, which details the assayed kinases, methodology, the respective phosphorylation sites, and the data (Array Report.pdf and Array Tables.xlsx in the Supplementary Materials).

### Real-Time PCR

RNA was isolated from the apex of the heart of Gα_s_ flx/flx cre^+^ and control mice after MI and AAB using the RNeasy kit (catalog number 74104 Qiagen). cDNA was synthesized using the High capacity cDNA reverse transcription Kit (4368814 Applied Biosystems). cDNA was quantified and 50 ng of cDNA/20 μL was used for the subsequent real time expression assay. Real-time PCR was performed using Taqman gene expression Assays (Applied Biosystems). By using Taqman gene expression assay (Assay ID: Mm01255747_g1-Nppa, Mm01255770_g1-Nppb, and Mm00600555_g1-Myh7) hypertrophy marker genes—*nppa, nppb*, and*myh7* were quantified. All genes were assayed in triplicates and relative gene expression was quantified using the comparative C_T_ method with *gapdh* as the reference housekeeping gene.

### Statistical Analysis

The statistical tests are indicated in the text or figure legends. Data are reported as mean ± SEM. and *P <*< .05*i >* was considered significant. Statistical analysis was carried out using the commercially available software, Prism version 8.0 (Graph Pad software, San Diego, California; www.graphpad.com).

## Results

The experimental protocols and timelines used in this study are summarized in Figure 1(B).

### Echocardiographic Measurement in Gα_s_ (flx, Flx) HCN4 Cre + Mice After Tamoxifen

**Figure 2.**
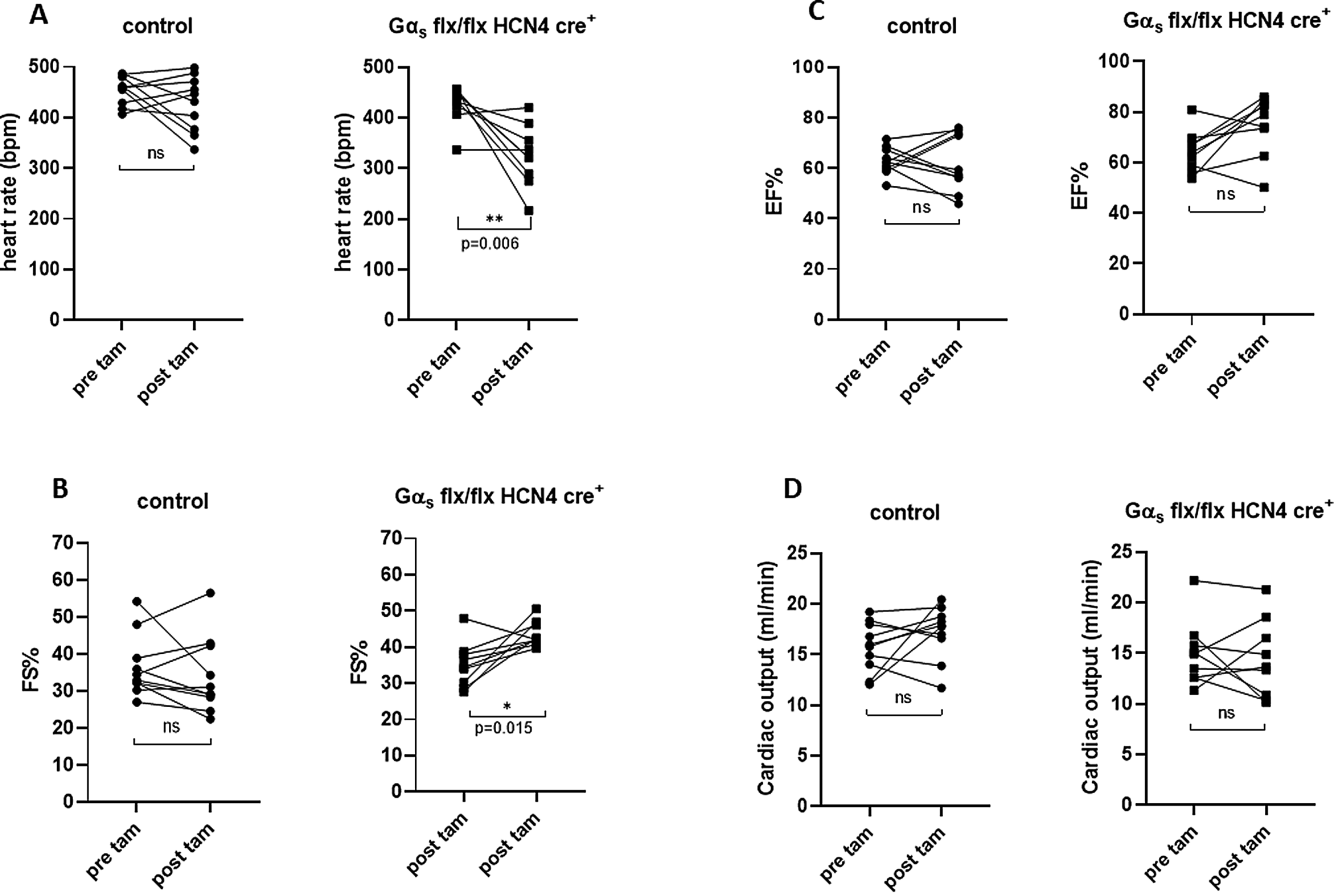
Echocardiographic parameters and deletion of Gα_s_. Echocardiographic parameters EF%, FS%, HR, and CO before and after the administration of tamoxifen in Gα_s_ (flx, flx) HCN4 cre + mice and littermate controls. (A) The administration of tamoxifen resulted in a fall in HR in Gα_s_ (flx, flx) HCN4 cre + mice but not in littermate controls **P = .006, *n* = 10 control, *n* = 9 Gα_s_ (flx, flx) HCN4 cre +. (B) FS% was found to be higher in Gα_s_ (flx, flx) HCN4 cre + mice *P = .015, *n* = 10 control, *n* = 9 Gα_s_ (flx, flx) HCN4 cre + analyzed using paired *t*-test. (C) and (D) EF% and CO were not altered.

We first examined the effects of deleting Gα_s_ in the SA node on contractile parameters measured by echocardiography. We measured these before and after the administration of tamoxifen in Gα_s_ (flx, flx) HCN4 cre + mice and littermate controls. The administration of tamoxifen resulted in a significant fall (20%) in HR in Gα_s_ (flx, flx) HCN4 cre + mice, but not in littermate controls (only 6%) consistent with our previous work (Figure 2A). Interestingly, at the slower HR there was a modest increase in FS (Figure 2C) but not a statistically significant increase in EF (Figure 2B) and no net change in CO (Figure 2D).

### Exercise Capacity

**Figure 3.**
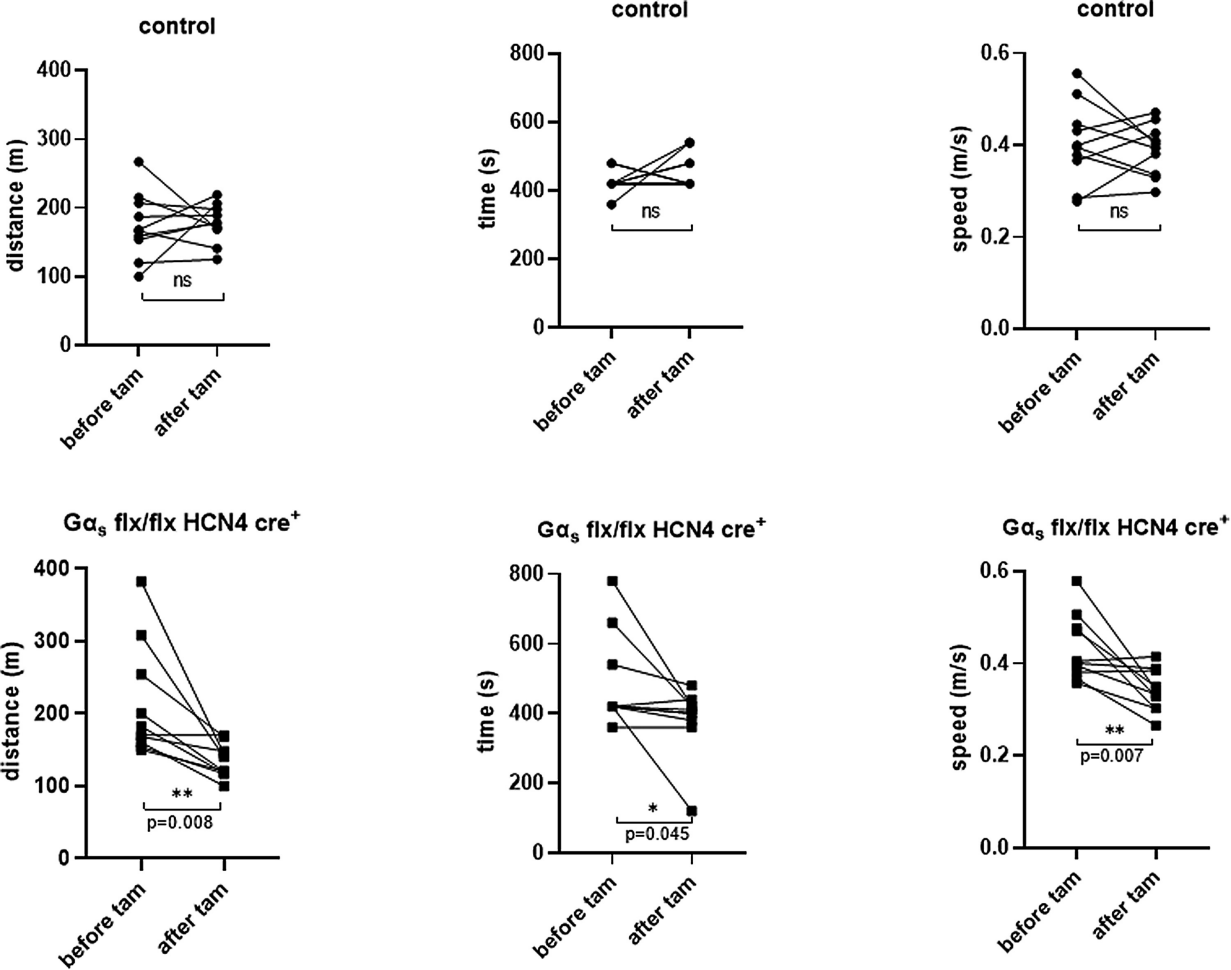
The performance with treadmill exercise. Upon exercise on a treadmill in contrast to control mice, Gα_s_ (flx, flx) HCN4 cre + mice ran a significantly shorter distance in less time at an overall slower speed. **P = .008, *P = .045, **P = .007, *n* = 10 control, *n* = 10 Gα_s_ (flx, flx) HCN4 cre + analyzed using paired *t*-test.

Using a treadmill, we assessed the capacity of the study mice to undertake physical exercise to exhaustion. We measured the total distance covered, the exercise time, and average speed. Littermate control mice were able to exercise to a similar level both before and after tamoxifen. In contrast, Gα_s_ (flx, flx) HCN4 cre + mice ran a shorter distance [136 m ± 7 Gα_s_ (flx, flx) HCN4 cre + and 177 m ± 9 control, *P* = .008] in less time [383 s ± 20 Gα_s_ (flx, flx) HCN4 cre + and 460 s ± 15 control, *P* = .04] at an overall slower speed [0.34 m/s ± 0.01 Gα_s_ (flx,  flx) HCN4 cre + and 0.39 m/s ± 0.01 control,  *P* = .007; *n* = 10 control, *n* = 10 Gα_s_ (flx, flx) HCN4 cre+; Figure 3)].

### Dobutamine Stress Test and Effects of IVB

**Figure 4.**
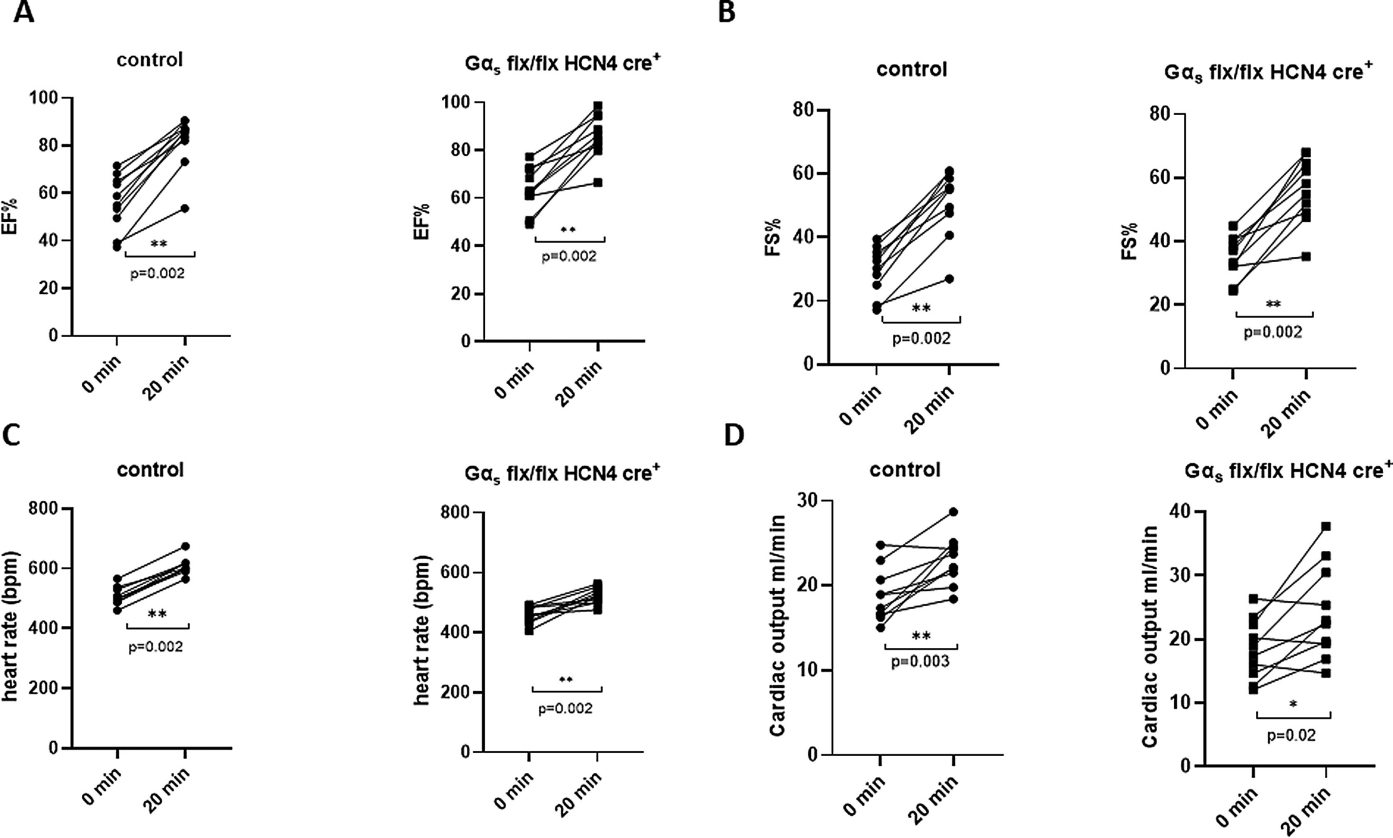
Dobutamine stress test. After i.p. injection of dobutamine the EF (A), FS (B), HR (C), and CO (D) all increased in Gα_s_ (flx, flx) HCN4 cre + and littermate control mice.**P = .002, *n* = 10 control, **P = .002, *n* = 10 Gα_s_ (flx, flx) HCN4 cre + for EF, FS, HR, and for CO **P = .003 for control and *P = .02 for Gα_s_ (flx, flx) HCN4 cre + analyzed using paired *t*-test.

**Figure 5.**
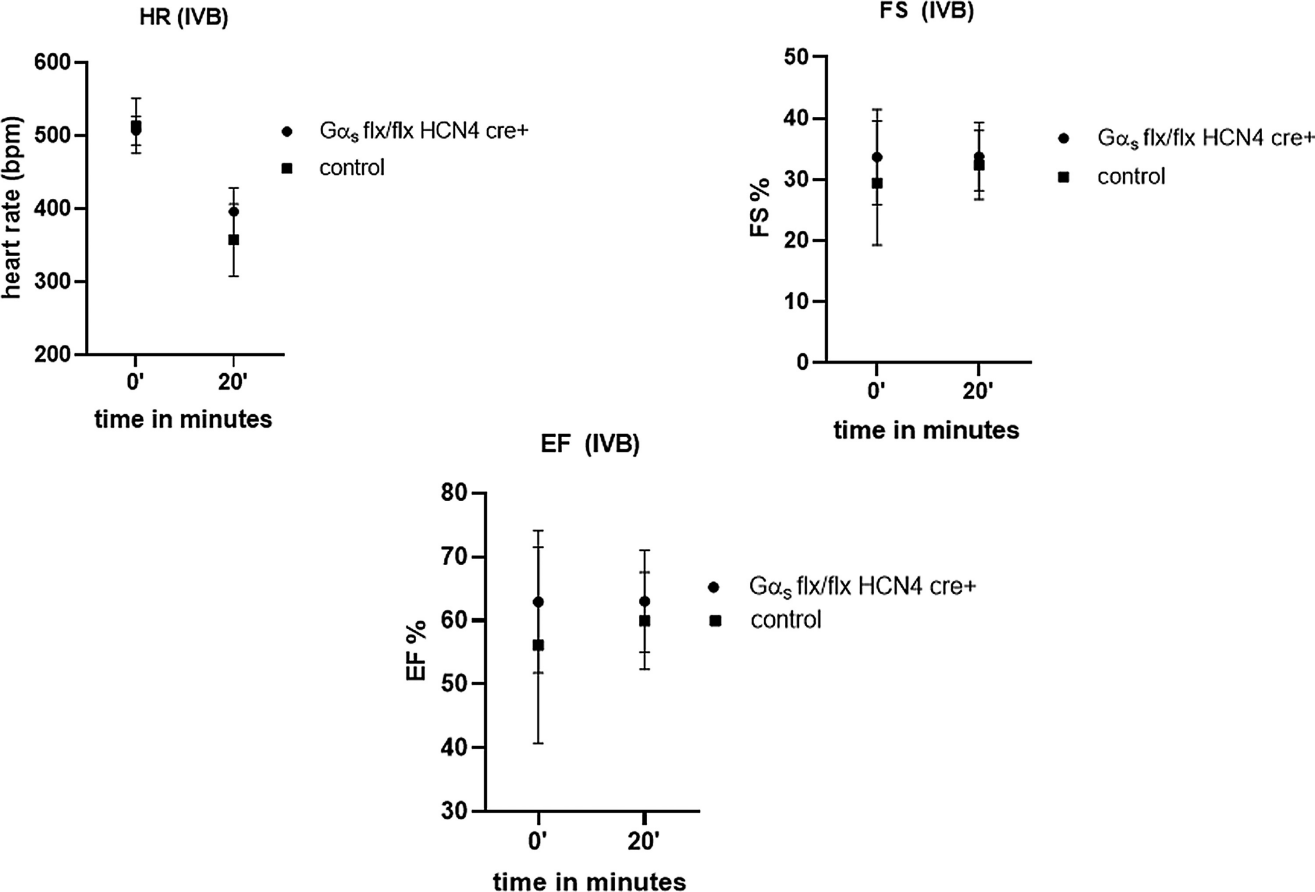
IVB administration. Following the administration of IVB the HR was reduced in both Gα_s_ (flx, flx) HCN4 cre + and littermate control mice after tamoxifen and there was no significant difference between the groups (*n* = 5 control and *n* = 5 Gα_s_ (flx, flx) HCN4 cre + mice).

We also assessed cardiac function using a dobutamine stress test. The EF, FS, HR, and CO all increased with i.p. injection of dobutamine in littermate control mice and the Gα_s_ (flx, flx) HCN4 cre + also showed increases in EF and FS (Figure 4). When comparing the responses between the genotypes only the HR response was different [0 min–507 bpm ± 8.9, 20 min–608 bpm ± 8.4 in control; 0 min–458 ± 8.3,  20 min–522 ± 8.0 in Gα_s_ (flx, flx) HCN4 cre+, *P*< .001 at both time points with unpaired *t*-test]. We also examined the HR lowering effect of IVB after tamoxifen and found no significant change in either control or Gα_s_ (flx, flx) HCN4 cre + mice (Figure 5). HR was lowered in both the groups and reached a plateau 20 min after the dosing. Other echocardiographic parameters were similar in both the groups after tamoxifen (EF and FS are shown Figure 5).

### Coronary Artery Ligation

**Figure 6.**
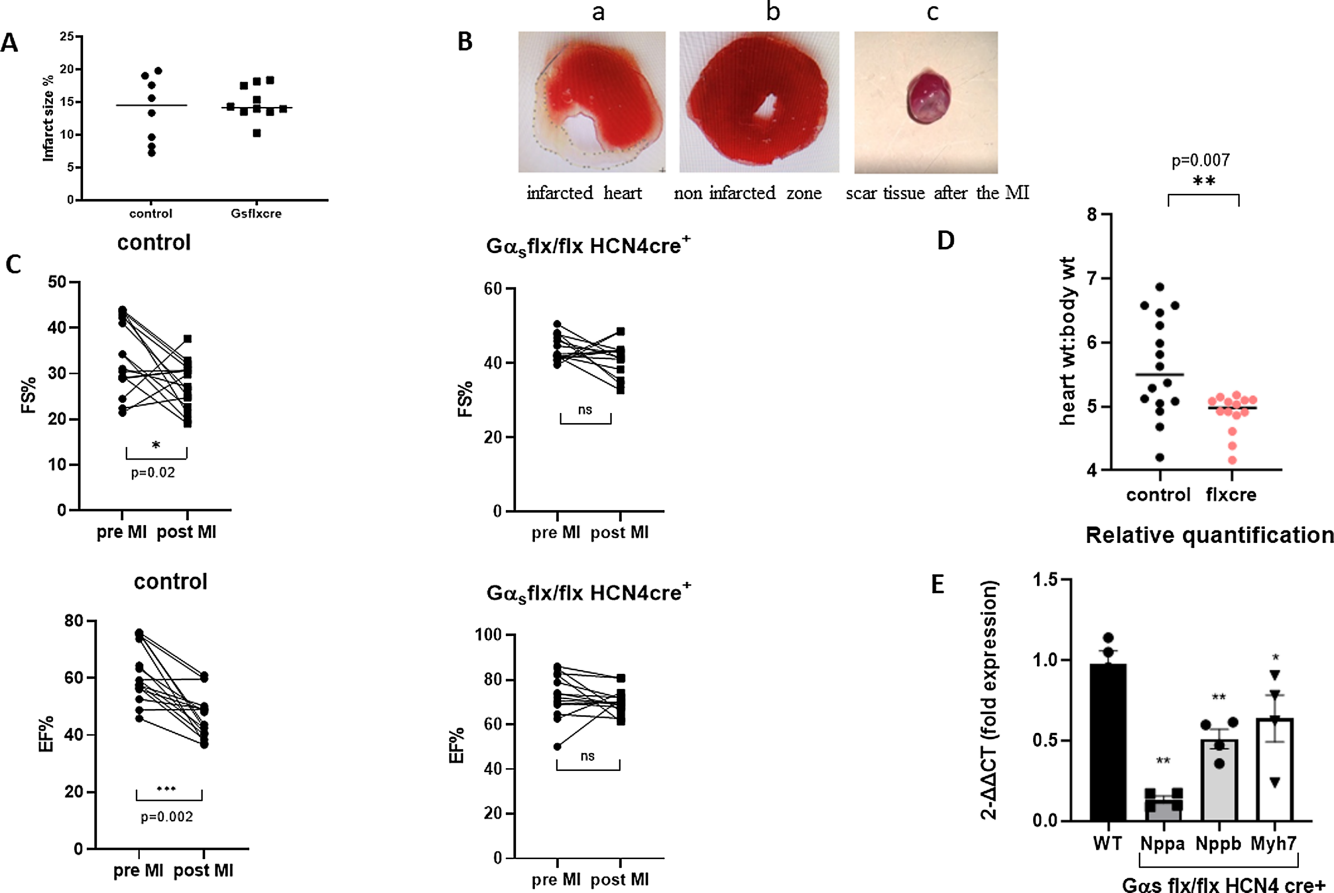
Coronary artery ligation. (A) Myocardial infarct size using TTC staining at the end of the study showed no difference between littermate controls (*n* = 8) and Gα_s_ (flx, flx) HCN4 cre + mice (*n* = 10). (B) Representative image of TTC staining: infarcted heart (a), noninfarcted zone (b) and showing scar tissue after the MI (c). (C) In littermate control mice after the coronary artery ligation, there was a reduction in FS *P = .02 and EF **P = .002 *n* = 16 control, *n* = 14 Gα_s_ (flx, flx) HCN4 cre +. (D) Increased heart weight to body weight ratio was observed in control mice. Data is shown as mean ± SEM **P = .007, *n* = 16 control, *n* = 14 Gα_s_ (flx, flx) HCN4 cre +. (E) Relative quantification of the hypertrophic marker genes *nppa,nppb*, and *myh7 (nppa* **P = .002, *nppb* **P = .002, and *myh7* *P = .02 *n* = 4 control *n* = 4 Gα_s_ (flx, flx) HCN4 cre+). (A), (D), and (E) analyzed using a Mann–Witney nonparametric *t*-test. (C) analyzed using paired *t*-test.

**Figure 7.**
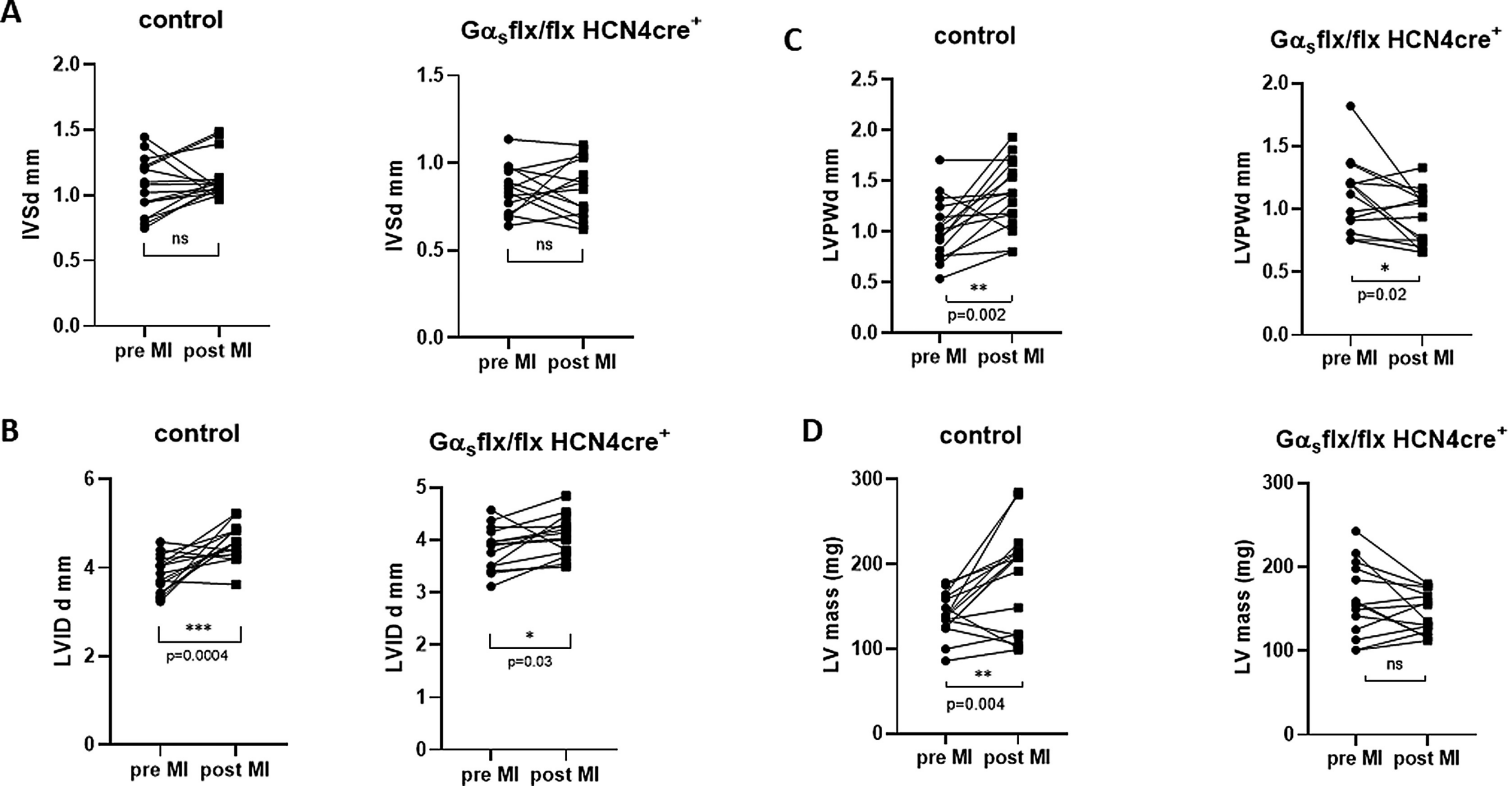
Wall dimensions after coronary artery ligation. (A) No difference between control and Gα_s_ (flx, flx) HCN4 cre + mice in IVSd. (B) Increased lumen depth was observed in control mice after MI compared to Gα_s_ (flx, flx) HCN4 cre + mice ***P = .0004 *n* = 16 control *n* = 14 Gα_s_ (flx, flx) HCN4 cre +. (C) LV wall thickness was also increased profoundly in control mice compared to Gα_s_ (flx, flx) HCN4 cre + mice **P = .002. (D) This hypertrophic response was also indicated by higher LV mass in the control mice but this did not occur in Gα_s_ (flx, flx) HCN4 cre + mice **P = .004 *n* = 16 control *n* = 14 Gα_s_ (flx, flx) HCN4 cre + analyzed using paired *t*-test.

We combined postmortem and echocardiographic measurements to examine pathological hypertrophy in the two models namely coronary artery ligation and aortic banding (see below). We first undertook coronary artery ligation of the left anterior descending coronary artery to mimic MI and subsequent impaired LV function. We performed echocardiography and we show EF and FS as indicators of LV function and LV posterior wall thickness at the end of diastole (LVPWd) and calculated LV mass for hypertrophy. Littermate control mice and Gα_s_ (flx, flx) HCN4 cre + mice were treated with tamoxifen and then underwent the surgical procedure. We assessed the size of the myocardial infarct using TTC staining at the end of the study and found there to be no difference between littermate controls and Gα_s_ (flx, flx) HCN4 cre + mice (Figure 6A). In littermate control mice 2 wk after the coronary artery ligation, there was a fall in EF (66.8% ± 2.2 pre MI and 48.4% ± 2.2 post MI, *P* = .002, *n* = 16) and a decline in FS (Figure 6C). However, in Gα_s_ (flx, flx) HCN4 cre + mice this did not occur 74.6% ± 1.9 pre MI and 70.2% ± 1.8 post MI, *n* = 14, no significant change). When comparing the genotypes, the EF was significantly lower postcoronary artery ligation in the control compared to the Gα_s_ (flx, flx) HCN4 cre + mice (*P*< .001, unpaired *t*-test). The hypertrophic marker genes expression [ratio of expression of Gα_s_ (flx, flx) HCN4cre + mice to control: *nppa* 2^-ΔΔCt = 0.14 ± 0.01,  *P* = .002; *nppb* 2^-ΔΔCt = 0.52 ± 0.12,  *P* = .002; and *myh7* 2^-ΔΔCt = 0.59 ± 0.13,  *P* = .01] were decreased in the left ventricle of Gα_s_ (flx, flx) HCN4 cre + mice compared to littermate controls (Figure 6E). Mice were sacrificed, and heart weight to body weight ratio was measured and was significantly increased in control mice [5.53 ± 0.21 control mice and 4.84 ± 0.09 Gα_s_ (flx, flx) HCN4 cre + mice; Figure 6D]. There was an increase LV chamber dimensions as indicated by an increase in LVIDd [4.73 mm ± 0.07 control mice and 4.04 mm ± 0.09 Gα_s_ (flx, flx) HCN4 cre + mice, *P* < .001, unpaired *t*-test]. LVPWd and LV mass increased after MI in control but not in Gα_s_ (flx, flx) HCN4 cre + mice. However, when comparing the genotypes directly post MI only the increase in LVPWd was significant [1.38 mm ± 0.08 control mice and 0.97 mm ± 0.04 Gα_s_ (flx, flx) HCN4 cre + mice, *P*< .001, unpaired *t*-test; Figure 7].

**Figure 8.**
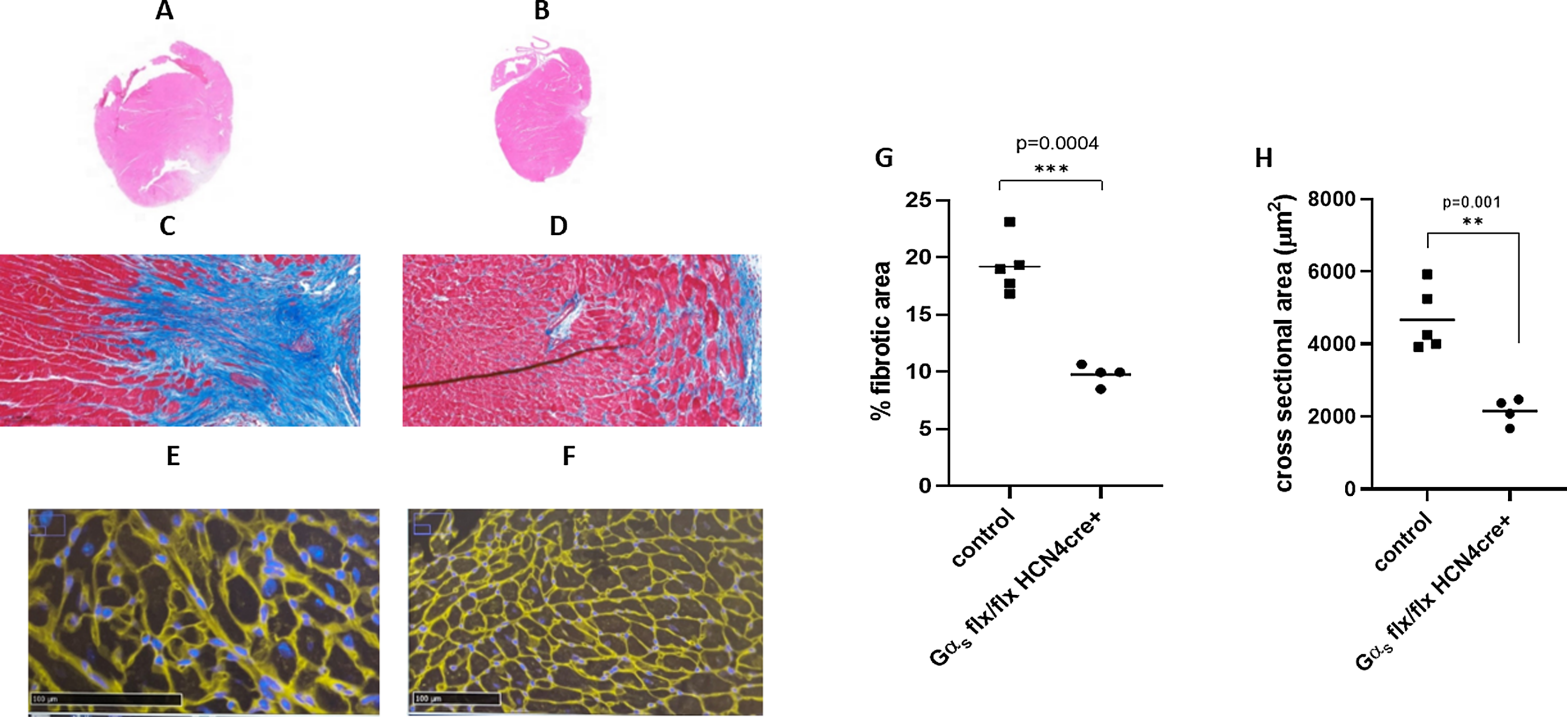
Histology by H&E, Trichrome and WGA staining. (A) and (B) Representative images of staining with H&E—control mice after MI showed an enlarged heart size compared to Gα_s_ (flx, flx) HCN4 cre + mice. (C) and (D) Sections of whole hearts stained with trichrome staining presented an increase in fibrotic tissue after MI in control mice than in Gα_s_ (flx, flx) HCN4 cre + mice (blue—collagen; red—myocardium magnification 20X). (E) and (F) WGA staining of ventricular sections of control and Gα_s_ (flx, flx) HCN4 cre + mice showed enlarged myocytes in control (scale bar 100 µm). (G) Fibrotic area analysis using Image J software shows increased fibrotic area in control mice [***P < .001 *n* = 5 control, *n* = 4 Gα_s_ (flx, flx) HCN4 cre + mice] using unpaired *t*-test. (H) Cross-sectional area analysis of WGA stained hearts using Image J also showed increased area in control mice [**P = .001 *n* = 5 control, *n* = 4 Gα_s_ (flx, flx) HCN4 cre + mice] using unpaired *t*-test.

We also performed cardiac histological analysis on the LV tissue away from the infarct on some of the mice. This shows increased cardiac size (Figure 8A top panel) in control mice [haematoxylin and eosin (H&E) stain] compared with that of Gα_s_ (flx, flx) HCN4 cre + mice (Figure 8B top panel). Trichrome staining revealed the extent of fibrosis away from the infarct site was greater in control mice [Figure 8C middle panel) compared to Gα_s_ (flx, flx) HCN4 cre + mice (Figure 8D middle panel]. WGA staining shows enlarged myocytes and increased extracellular matrix in control (Figure 8E bottom panel) compared to Gα_s_ (flx, flx) HCN4 cre + mice (Figure 8F bottom panel). Quantification confirmed fibrosis was in control 19.2 ± 1.1% mice compared to Gα_s_ (flx, flx) HCN4 cre + mice 9.70 ± 0.36% (*P*< .001, Figure 8G). Quantification of cross-sectional area myocytes revealed increased area in control mice 4672 μm^2^ ± 392 compared to Gα_s_ (flx, flx) HCN4 cre + mice 2155 μm^2^ ± 160 (*P* = .001, Figure 8H) indicative of cellular hypertrophy.

### Abdominal Aortic Banding

**Figure 9.**
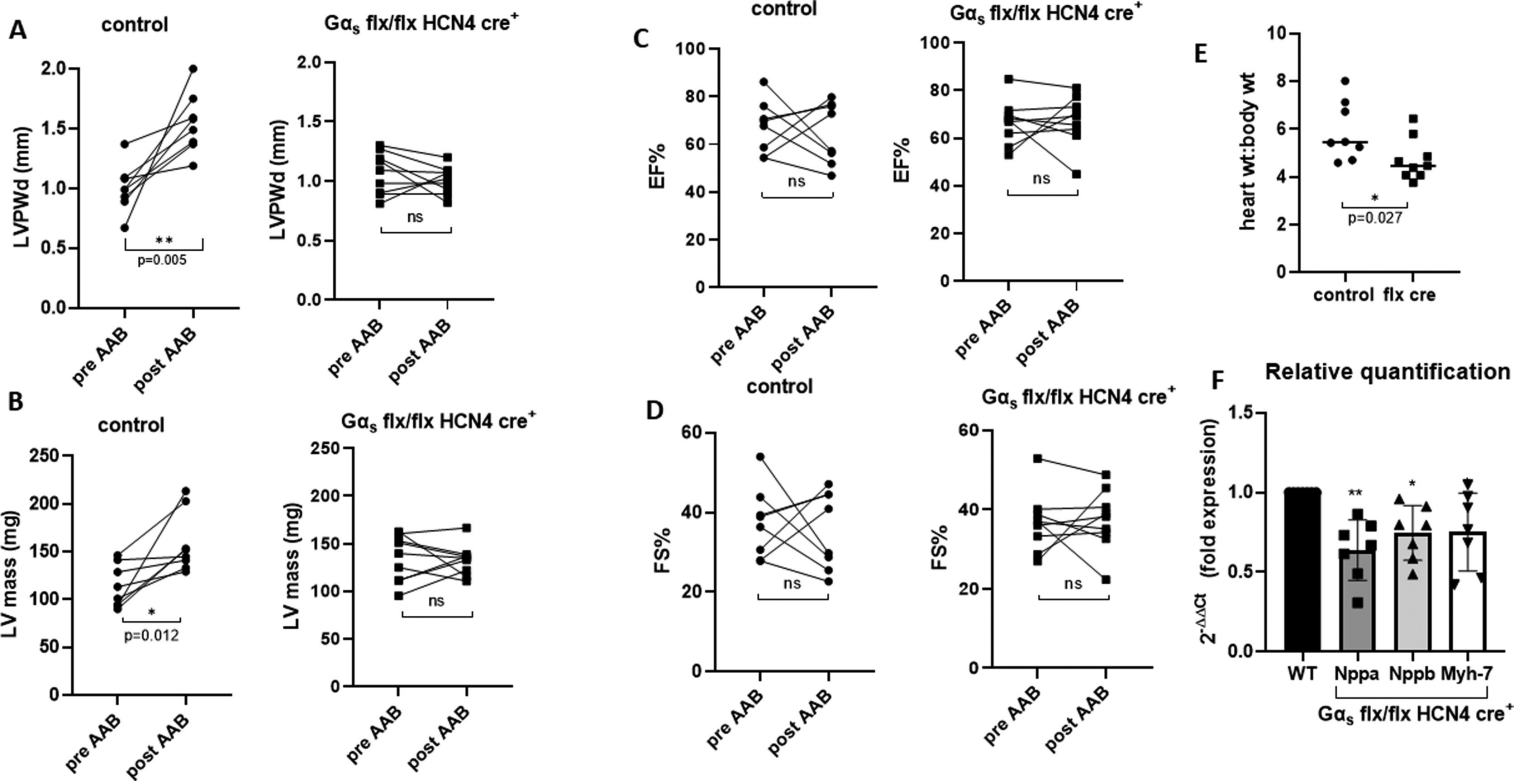
Abdominal aortic banding. (A) In littermate control mice 2 wk after abdominal aortic banding, this indeed led to a modest increase in LVPWd **P = .005 and (B) LV mass *P = .015 *n* = 8 control, *n* = 9 Gα_s_ (flx, flx) HCN4 cre + with echocardiography. (C) EF and (D) FS were not changed analyzed by Wilcoxon matched paired nonparametric *t*-test. (E) This was confirmed by measuring the ratio of heart weight to body weight at the end of the study, which showed an increase in ratio in littermate control mice *P = .027, *n* = 8 control, *n* = 9 Gα_s_ (flx, flx) HCN4 cre +. In (E), HW: BW is shown as mean ± SEM, analyzed using Mann–Witney nonparametric *t*-test. (F) Relative quantification of hypertrophic marker genes *nppa* and *nppb showed* increased expression in control mice compared to Gα_s_ (flx, flx) HCN4 cre + mice *nppa* **P = .002, *nppb* *P = .03 *n* = 7 control, *n* = 7 Gα_s_ (flx, flx) HCN4 cre + mice analyzed by Mann–Witney nonparametric *t*-test.

In addition, we explored a second murine model of cardiac hypertrophy. We performed abdominal aortic banding above the renal arteries, which results in hypertension and compensatory LV hypertrophy. We examined the mice 2 wk after the surgery so that there was a predominantly hypertrophic response without impaired LV function. In littermate control mice, LVPWd and LV mass increased whereas this did not occur in Gα_s_ (flx, flx) HCN4 cre + mice (Figure 9A and B). When comparing the genotypes the increase in LVPWd was statistically significant [1.5 mm ± 0.08 control mice and 1.00 mm ± 0.03 Gα_s_ (flx, flx) HCN4 cre + mice, *P*< .001, unpaired *t*-test] and the difference in LV mass almost significant [158.6 mg ± 10.5 control mice and 132.6 mg ± 5.0 Gα_s_ (flx, flx) HCN4 cre + mice], *P* = .05, unpaired *t*-test; Figure 9A and B). EF and FS were not changed consistent with preserved contractile function (Figure 9C and D). The ratio of heart weight to body weight was measured at the end of the study [5.9 ± 0.41 control mice and 4.68 ± 0.27 Gα_s_ (flx, flx) HCN4 cre + mice, *P* = .02; Figure 9E] and also hypertrophic marker gene expression (*nppa* 2^-ΔΔCt = 0.63 ± 0.06 *P* = .002; *nppb* 2^-ΔΔCt = 0.74 ± 0.05 *P* = .03; and *myh7* 2^-ΔΔCt = 0.75 ± 0.07,  not significant, in Gα_s_ flx/flx) HCN4 cre + mice and control; Figure 9F).

### Phospho-Antibody Array

**Figure 10.**
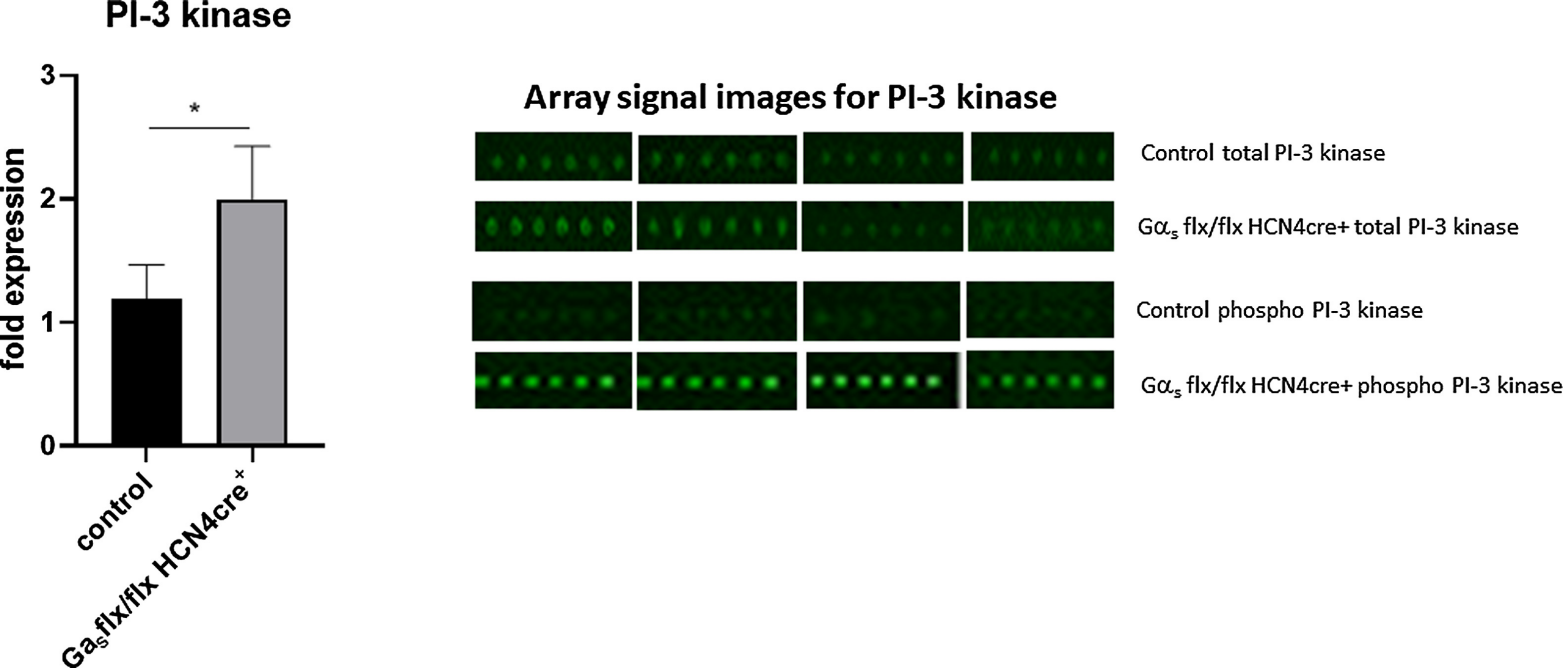
Phospho-antibody array. Protein kinase activation was measured using phospho-specific antibodies and the results expressed as a ratio of the signal from the phospho-antibody with that of the total protein antibody. The ratio of the signal comparing PI3-kinase p85-subunit alpha/gamma (phospho-Tyr467/Tyr199) to PI3-kinase p85-subunit alpha/gamma (Ab-467/199) activation was increased approximately 2-fold in Gα_s_ (flx, flx) HCN4 cre + mice (*n* = 4, median taken of 6 biological replicates shown) compared to littermate controls (*n* = 4, median taken of 6 biological replicates shown). Array signal intensity shows it has increased in AAB Gα_s_ (flx, flx) HCN4 cre + mice compared to control. Data is shown as mean ± SEM, *P << .05*i>, n* = 4 control, *n* = 4 Gα_s_ (flx, flx) HCN4 cre + using Mann–Witney nonparametric *t*-test.

In order to assess potential protein kinase pathways that may be activated in the left ventricle by relative bradycardia, we used a phospho-antibody array (see “Methods”). The array profiles a large number of protein kinases by comparing the ratio of phosphorylated protein kinase to total protein kinase. The pdf and excel spreadsheet provided by the company are provided as Supplementary Materials, which details the assayed kinases, methodology, the respective phosphorylation sites, and the data (Array Report.pdf and Array Tables.xlsx in the Supplementary Materials). LV lysates were prepared from the hearts of Gα_s_ (flx, flx) HCN4 cre + mice and littermate controls at 2 wk after the mice had undergone abdominal aortic banding. Protein kinase activation was measured using phosphospecific antibodies and the results expressed as a ratio of the signal from the phosphoantibody with that of the pan-protein antibody. A large number of candidates were tested (see Supplementary Materials) and the only one with a significant change in activity was PI-3 kinase (Figure 9). The ratio of the signal comparing phosphorylated PI3-kinase p85-subunit alpha/gamma (Phospho-Tyr467/Tyr199) to PI3-kinase p85-subunit alpha/gamma (Ab-467/199) was increased approximately 2-fold in Gα_s_ (flx, flx) HCN4 cre + mice compared to littermate (Figure 10). We also examined whether PI-3 kinase or any other protein kinases using the same phosphoprotein array were activated prior to AAB in the Gα_s_ (flx, flx) HCN4 cre + after tamoxifen, but we observed no changes in relative activity of any of the kinases assayed compared to littermate controls (not shown).

## Discussion

In this study, we use a genetic model^13^ to explore the role of HR reduction in physiological and pathological processes. As expected, exercise capacity is impaired by deletion of Gα_s_ in the SA node. However, in models of MI and increased afterload, the bradycardia consequent upon deletion of Gα_s_ in the SA node attenuated pathological hypertrophic responses in both models.

### HR and Physiological Function

After tamoxifen, HR fell in the mutant mice as expected with a modest increase of FS, though the increase in EF was not statistically significant. The net effect of these changes was that CO remained relatively preserved at rest in the mutant mice. These changes did not occur in littermate control mice. This is consistent with a negative force–frequency relationship that is known to be present in murine hearts.^31^ Interestingly, in man, there is a positive relationship in the healthy left ventricle but this remodels in heart failure to a negative one^32^ and has been suggested to be one of the beneficial effects of HR reduction in heart failure.^33^ However, when undertaking treadmill exercise the Gα_s_ (flx, flx) HCN4 cre + mice after tamoxifen showed reduced exercise ability compared to littermate controls. This was consistent with results from a dobutamine echocardiographic stress test, which showed the HR at rest being lower and then a smaller increase with dobutamine. This is consistent with our previous results of an impaired but not abolished chronotropic response to isoprenaline in awake conscious mice.^13^ The results are consistent with a major adaptive response to exercise being an increase in HR and loss of this leads to a likely survival disadvantage in the face of predation.

### HR and Pathological Cardiac Hypertrophy

There has been a renewed interest in the disease-modifying effects of selectively reducing HR without modifying LV contractile performance. Much of this has been driven by clinical trials involving the administration of IVB.^5^ In the setting of heart failure, the SHIFT study demonstrated that in those patients with LV EF of less than 35% and a HR above 70 beats per minute, the addition of IVB to standard therapy resulted in a close to 20% reduction in hospitalization and death from heart failure.^7^ Furthermore, the improvement in outcome correlated with the degree of HR reduction.^2^ The results were not so convincing in the BEAUTIFUL trial.^34^ Our results in the murine model are consistent with a selective effect of HR reduction on pathological hypertrophic responses that are a prominent maladaptive feature of heart failure.

Animal models and human tissue studies have shown that various HCN isoforms (HCN1, 2, and 4, depending on the species studied) are upregulated in the atria and ventricles in heart failure as part of the reversion to an embryonic expression profile.^9,10,12,35^ Thus, a direct block by IVB of If in the ventricle may attenuate hypertrophy, apoptosis, and electrophysiological remodeling.^36^ One major advantage of the approach we have undertaken is that it disentangles the effects of HR reduction from the other effects of IVB. Indeed, we saw no effects of IVB on contractile function in Gα_s_ (flx, flx) HCN4 cre + mice and littermate controls after tamoxifen. Furthermore, we administer tamoxifen before implementation of the abdominal aortic banding and coronary artery ligation models, thus overcoming potential ectopic ventricular expression of cre recombinase from the HCN4 promoter. Resting HR is a heritable trait and there have a number of genome-wide association studies revealing many loci associated with the phenotype.^37–39^ As more genetic variants are discovered, Mendelian randomization and other genetic approaches become a viable method to examine the causality of HR in cardiometabolic pathology. It is known that a genetic risk score derived from HR-related loci is correlated to all-cause mortality.^38^ Furthermore, Mendelian randomization studies show potential causality between higher resting HR and type II diabetes mellitus and other cardiometabolic traits.^40^ The analysis of an association between resting HR and outcome in heart failure or in coronary artery disease has not yet been performed.

### How Is HR Reduction Beneficial?

HR reduction can have a number of (patho)physiological effects. A slower HR leads to decreased oxygen consumption and increased time during diastole for coronary perfusion^41^ and likely accounts for the efficacy in preventing angina. As mentioned above, HR reduction may paradoxically improve contractility in the failing heart.^32^ In a rat model of heart failure following coronary artery ligation, cardiac metabolism and function were improved after the administration of IVB.^42^ Our results showing an attenuation of hypertrophic responses in 2 murine models are certainly compatible with these studies.

However, what has not been addressed is what the potential cell signaling pathway, if any, might be responsible for the improvement. It is known that the LV transcriptome is not modified substantially by HR reduction and, thus we focussed on potential activation of protein kinases.^43^ The use of in vivo animal models is not the easiest way to explore this, but we performed some preliminary investigations. We used a phosphoantibody array that included over 220 specific kinases. The only protein kinase that was differentially activated was phosphoinositide 3-kinase. The antibody detects both activated alpha and delta isoforms, but it is known that phosphoinositide 3-kinase alpha is highly expressed in cardiomyocytes.^44^ Classically, phosphoinositide 3-kinase alpha engagement occurs after insulin receptor and\or insulin like growth factor receptor stimulation.^45^ It is known that insulin receptor stimulation is cardioprotective likely mediated to a large extent via a phosphoinositide 3-kinase alpha and the consequent metabolic adaptations.^45^ Phosphoinositide 3-kinase alpha is known to be important in determining cardiomyocyte size and in maintaining contractility through downstream actions on akt protein kinase.^46^ Furthermore, direct activation of phosphoinositide 3-kinase alpha is known to be protective in heart disease. For example, genetic reduction of phosphoinositide 3-kinase alpha activity impairs contractile function, exacerbates pathological hypertrophy, and worsens heart failure in various murine models.^47–49^ Thus, our observations of enhanced phosphoinositide 3-kinase alpha activity are entirely consistent with a body of literature showing cardioprotective effects for this protein kinase.

It is not clear what the upstream mechanism might be, but this would be more easily delineated in future studies in a cellular model. There are known to be interactions between mechanical stress signaling pathways and phosphoinositide 3-kinase alpha activation.^50,51^ It is also plausible that insulin-like growth factors could be produced in areas of tissue damage in cardiomyocytes.^52^ Interestingly, the activation of phosphoinositide 3-kinase only occurs when HR slowing is combined with a pathological stimulus such as abdominal aortic banding. Limitations of our study are that we did not examine whether phosphoinositide 3-kinase activation also occurred in the MI model and have not examined a more extended time window after the pathological challenge.

In summary, we have used a unique genetic model to explore the influence of bradycardia on cardiac function. As would be expected the relative impairment of the chronotropic response during exercise leads to impaired performance. However, in the pathological setting, bradycardia attenuates pathological cardiac hypertrophy possibly through the activation of phosphoinositide 3-kinase.

## Funding

This work was supported by the British Heart Foundation (RG/15/15/31742) and 1R01HL146514-01A1 from the National Institute of Health (USA), partially by the Intramural Research Program of the National Institute of Diabetes and Digestive; and Kidney Diseases at the National Institutes of Health and was facilitated by the National Institute for Health Research, Cardiovascular Biomedical Research Centre at Barts.

## Supplementary Material

zqac055_Supplemental_Files

## Data Availability

The data and resources underlying this article will be shared on reasonable request to the corresponding author.
