# Supplementary material for: Slowing Heart Rate Protects Against Pathological Cardiac Hypertrophy
Source: Function (Oxf). 2022 Nov 1;4(1):zqac055. doi: 10.1093/function/zqac055 (PMC9761894; doi:10.1093/function/zqac055)
Supplement: zqac055_Supplemental_Files [file zqac055_supplemental_files.zip › Array Report.docx]

Assay Data Analysis

Array layout: array layout on the slide.

Ab list: antibody list with SwissProt number.

Untreated: raw data (GPR file from scanner) of untreated sample.

Treated: raw data (GPR file from scanner) of treated sample.

Untreated average and treated average:

- For each spot on the array, median signal intensity is extracted from array image.
- For each antibody, using the median signal intensity (F532 Median), the average signal intensity of the replicate spots is calculated. The data is labeled as Average Signal Intensity of Replicate Spots on the Array.
- The CV of the Replicates on the Array is the coefficient of variation for the replicate spots for each antibody.
- SNR 532: signal to noise ratio defined by (spot mean – noise mean)/(standard deviation noise).
- Analysis of images allows to exclude some signals from the average calculation due to stain or high background on the spot (Cell of worksheet is highlighted in yellow).

Assay data:

- For normalization, within each array slide the median value of the Average Signal Intensity for all antibodies on the array is determined. This value is presented as Median Signal.
- Normalized data = Average Signal Intensity of Replicate Spots / Median Signal.
- The results are as labeled as Data Normalized to Median Signal.
- Using the normalized data, the fold change between control and treated samples is determined :
- Fold change = Treated/Control
- Results are highlighted as:
- Red: increase in expression (ratio ≥ 2)
- Green: decrease in expression (ratio ≤ 0.5)
- The cutoff for the true signal can be selected in cell E3 (Cf 5. Data interpretation/True signal for more explanation).

Protein sorting:

- Each protein and corresponding phospho protein are sorted in the same row.
- In some cases, paired antibodies do not exist. Then, you may substitute the signal of site-specific antibody for another. In the example below, you may use the signal of CaMK2(Ab-286) as the signal for total protein of CaMK2.

| CaMK2 (Phospho-Thr286) |  | CaMK2 (Ab-286) |
| --- | --- | --- |
| CaMK2 (Phospho-Thr305) |  | Use signal of CaMK2 (Ab-286) |

- In case of substitution, the antibody is highlighted in yellow.
- If an antibody is not available, the cell of worksheet is highlighted in grey.
- Using the Average Signal Intensity of Replicate Spots on the Array, for each pair of phospho-antibody and non-phospho-antibody, the Signal Ratio of Phospho-protein to Non-phospho-protein is determined.
- Ratio = (Average Signal Intensity of Phospho-Antibody)/(Average Signal Intensity of Non-Phospho-Antibody)

P to N Ratio:

- P/N Ratio Fold change = Treated/Control
- Results are highlighted as:
- Red: increase in expression (ratio ≥ 2)
- Green: decrease in expression (ratio ≤ 0.5)

Data Interpretation

Fold change between samples

- In general, a fold change is considered significant when the value is less than 0.5 or greater than 2. A value of 0.5 indicates that the protein amount has decreased by 50%, and a value of 2 means the protein amount has doubled.
- However, the cut-off value for significant fold change can vary with sample type, treatment method and dosage, and other aspects of the experiment. You should determine the appropriate cut-off accordingly.

Phospho antibody vs. Non-phospho antibody (Phospho arrays only)

- In the phospho antibody arrays, there are two types of antibodies, phospho antibodies and non-phospho antibodies, such as p53 (Phospho-Ser15) and p53 (Ab15). The number indicates phosphorylation site. For instance, antibody p53 (Ab-15) is made from a synthetic nonphosphopeptide derived from human p53 around the phosphorylation site of Serine 15. It detects endogenous levels of total p53 protein. Antibody p53(Phospho-Ser15) is made from a synthetic phosphopeptide derived from human p53 around the phosphorylation site of Serine 15. It detects endogenous levels of p53 only when phosphorylated at Serine 15. In most cases, both phosphospecific antibodies and their non-phospho pairs are included in the array.
- There may be multiple non-phospho antibodies against the same protein. They all detect the same total protein but each recognizes specific residues around the corresponding phosphorylation site. For example, c-Jun (Ab-91) detects total c-Jun protein by recognizing specific residues around phosphorylation site threonine 91; cJun (Ab-170) detects total c-Jun protein by recognizing specific residues around phosphorylation site tyrosine 170.

True signal (ie SNR 532)

- When evaluating the data with significant fold changes, it is important to go back and review the signal intensity, and make sure the data is derived from true signals.
- A signal is considered a true signal when the signal intensity is at least 3 times of the background intensity value.
- Typically the signal intensity of Empty Spots can be used as a measure of the background intensity.

Data analysis method

- There are many different ways to analyze antibody array results. Some methods may produce more meaningful results than others. The method used here is just one of the methods, but it may not be the optimal choice for your sample type and experimental settings. We encourage you to take the signal intensity data and analyze them using other methods.

Proteins not detected

- Because the proteins used in the antibody array assays are not denatured, their tertiary folding structures are intact. As a result, there is an increased chance for false negatives due to inaccessible binding residues.
- Certain proteins form complexes with other proteins immediately after phosphorylation. This phenomenon can block the phosphorylation site from being recognized by the antibodies; as a result, the protein is not detected.

Result verification

- Protein changes derived from antibody array assays should be validated by other methods, such as immunoblotting. Most of the changes can be reproduced by Western blots, but some may not due to the dependency of non-denaturing conditions in antibody arrays.

Array images


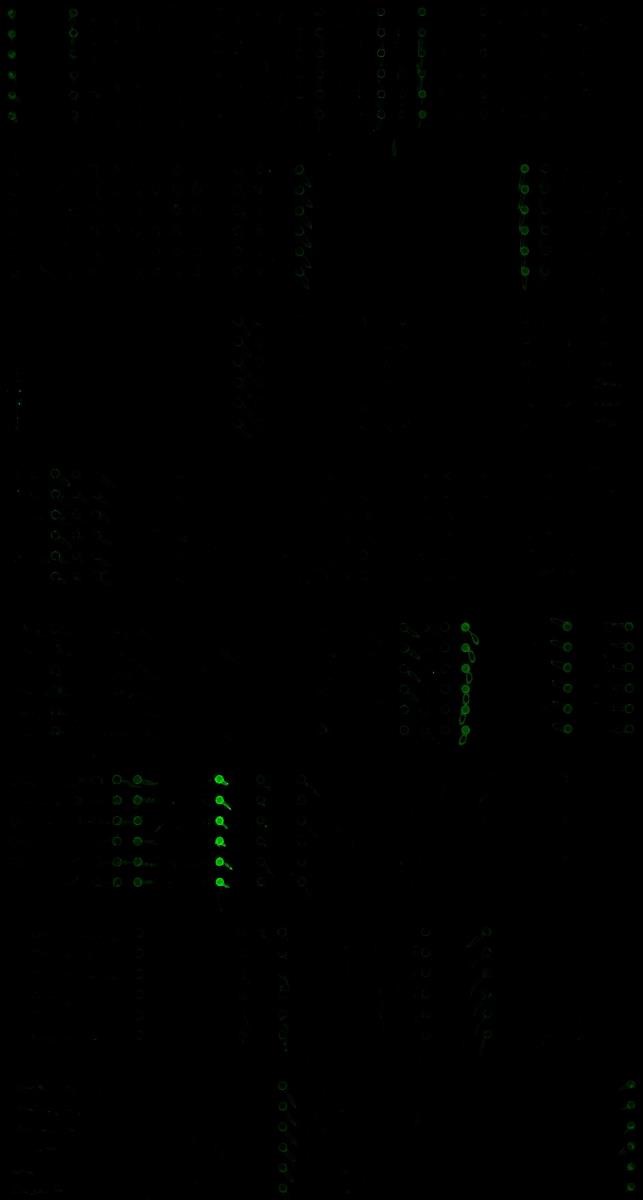

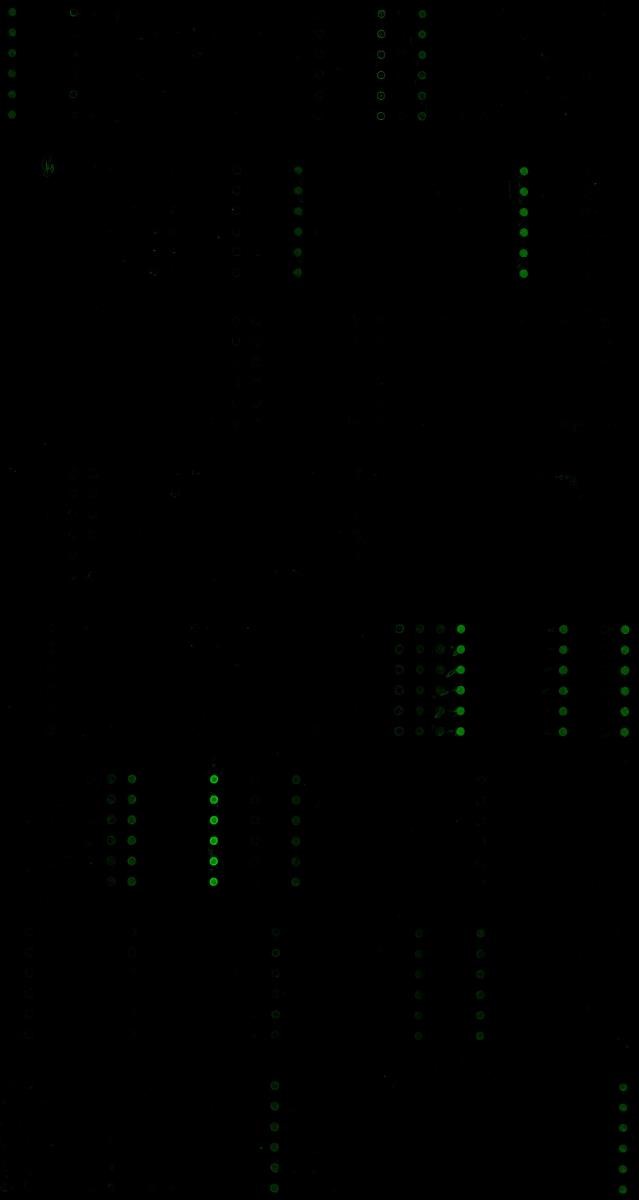


Slide 4000031988 Slide 4000031978

Sample 1_5F flxcre+ (KO) Sample2_3F flxcre- (Control)


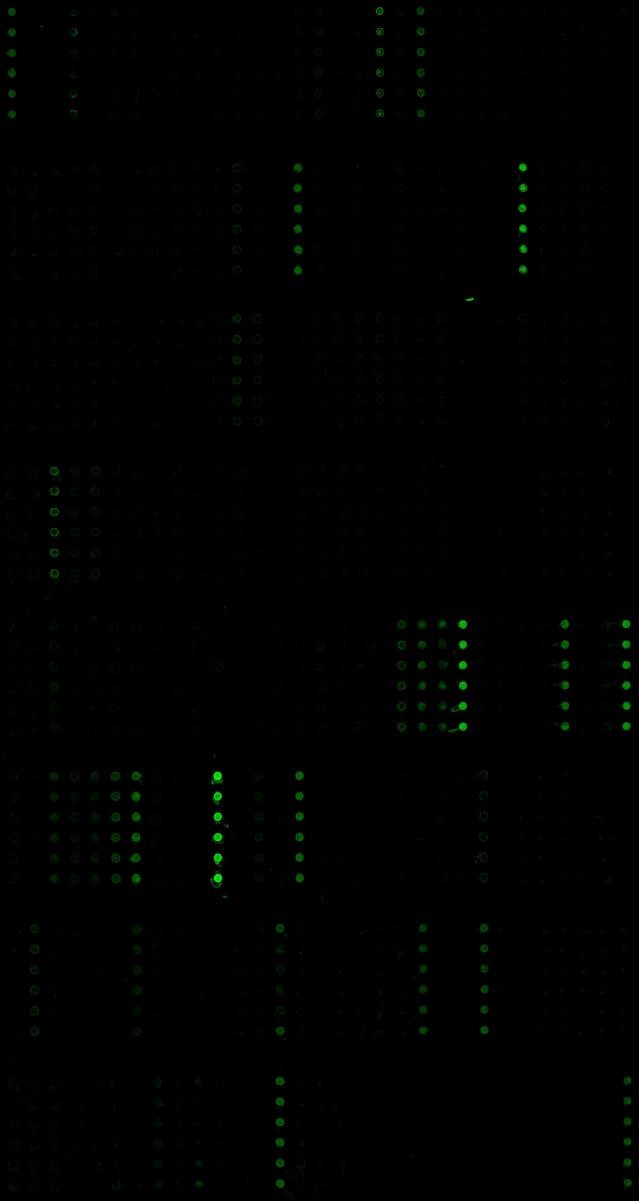

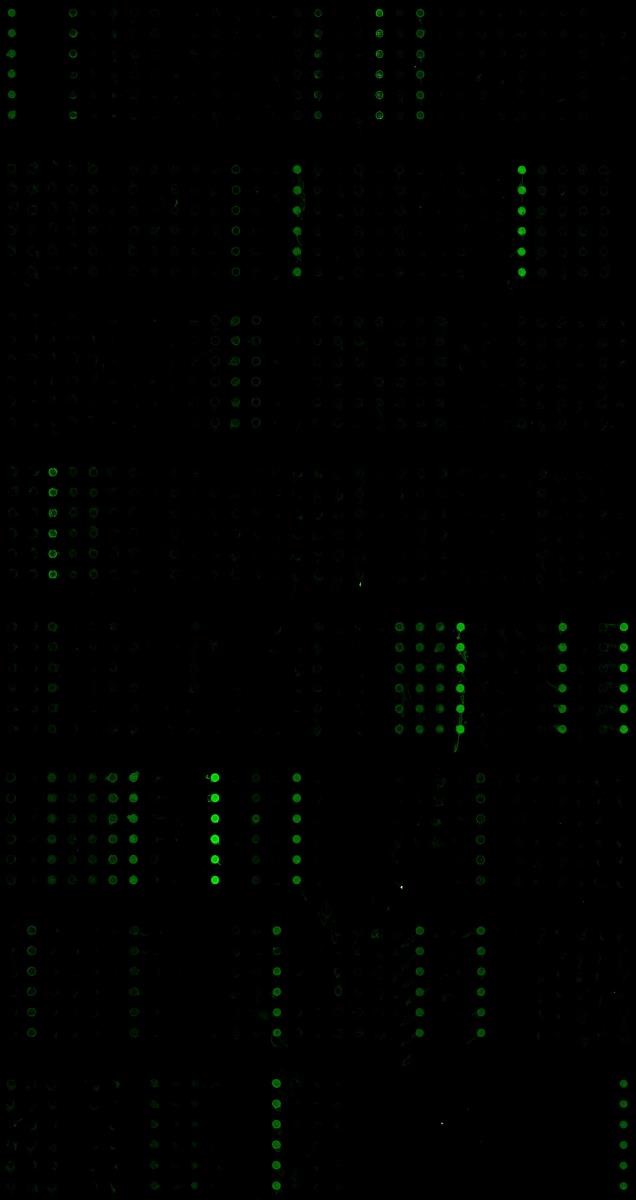


Slide 4000031977 Slide 4000031976

Sample 3_6F flxcre+ (KO) Sample 4_2F flxcre- (Control)


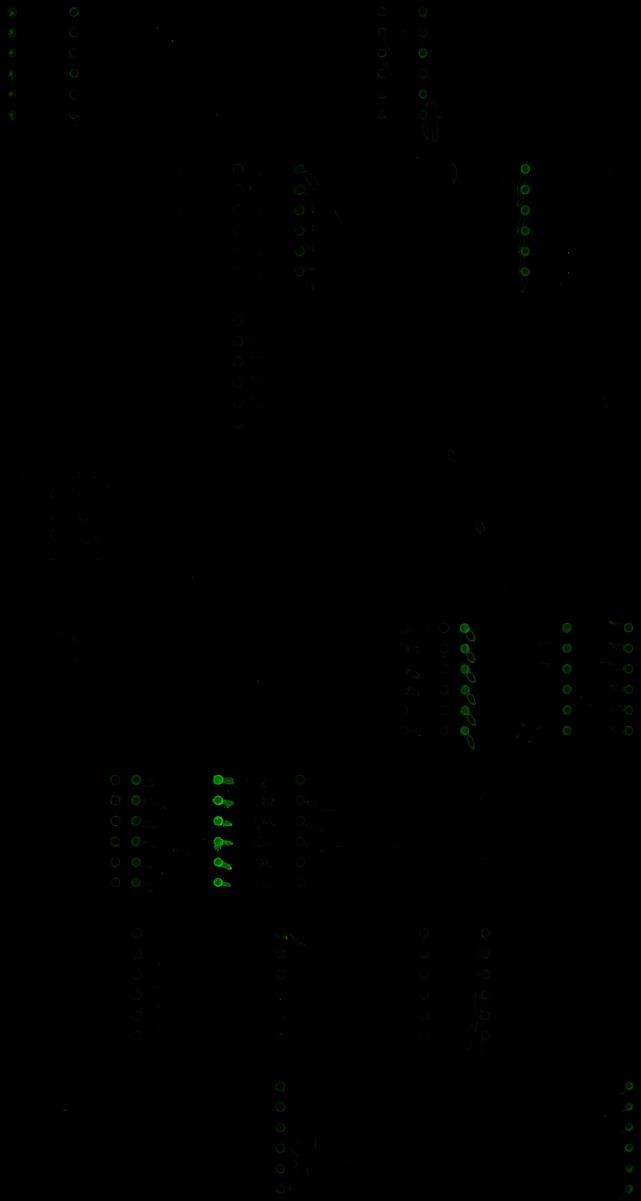

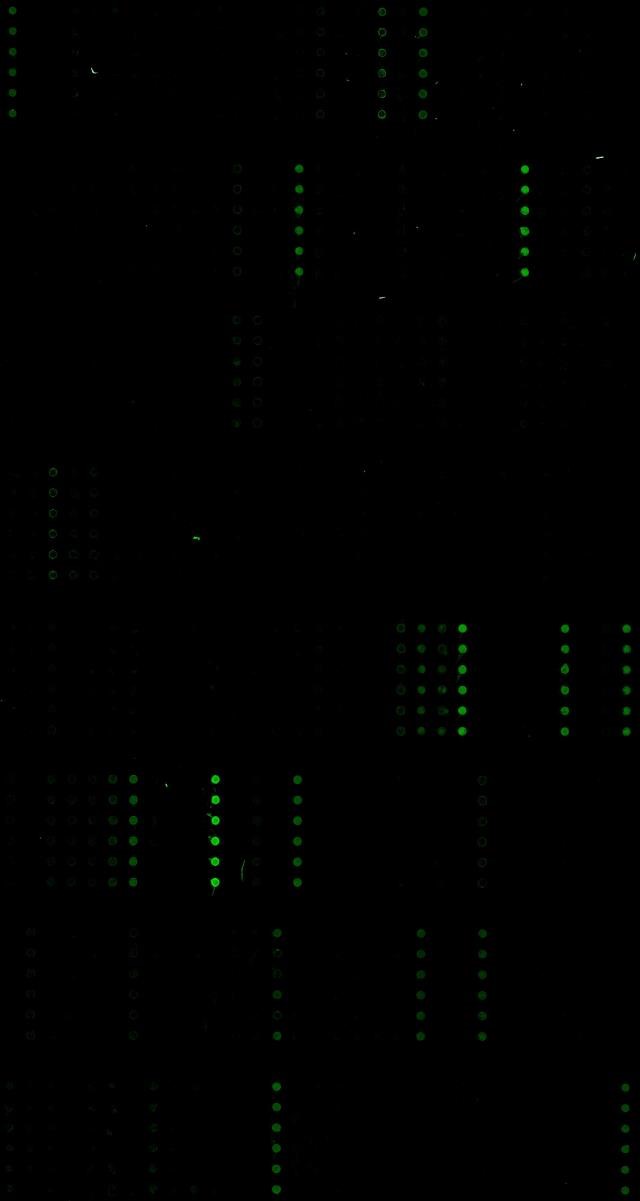


Slide 4000031987 Slide 4000031980

Sample 5_1M flxcre+ (KO) Sample 6_2F flxcre- (Control)


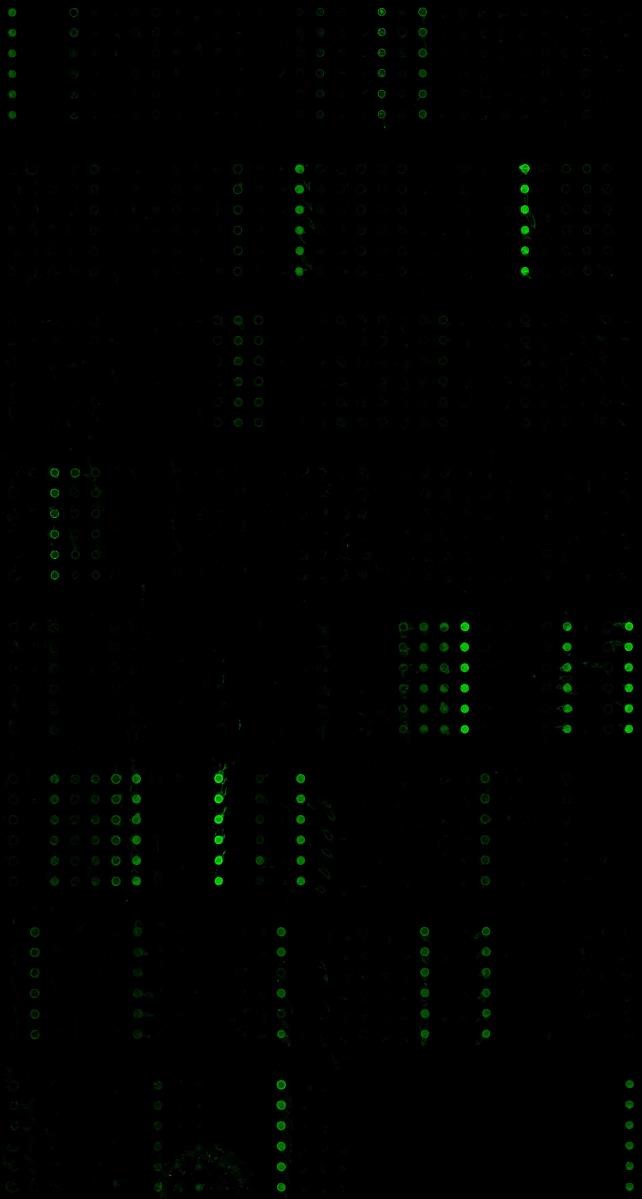

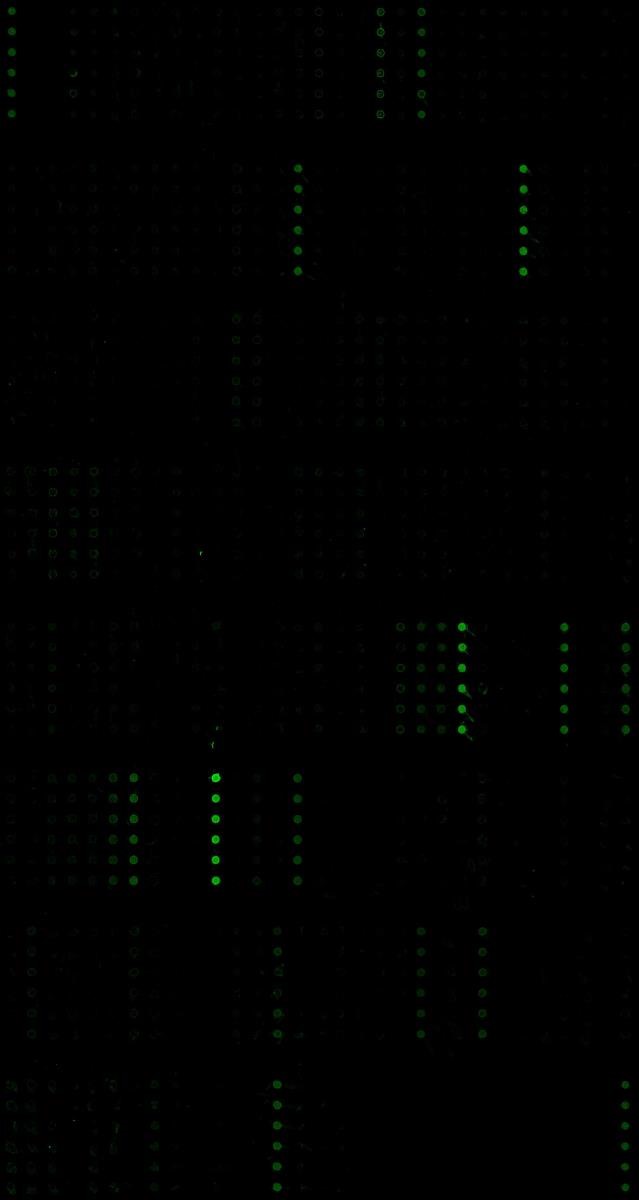


Slide 4000031975 Slide 4000031979

Sample 7_2M flxcre+ (KO) Sample 8_3F flxcre- (Control)
